# Effect of Space Allowance on Pig Performance, Carcass Traits and Meat Quality in Italian Heavy Pigs Reared Under Two Housing Systems

**DOI:** 10.3390/foods14162817

**Published:** 2025-08-14

**Authors:** Paolo Ferrari, Andrea Bertolini, Anna Garavaldi, Valerio Faeti, Monica Bergamaschi, Cecilia Loffi, Anna Pinna, Roberta Virgili

**Affiliations:** 1 CRPA, Research Centre for Animal Production s.c.p.a., 42121 Reggio Emilia, Italy; a.bertolini@crpa.it (A.B.); a.garavaldi@crpa.it (A.G.); 2 CREA, Council for Agricultural Research and Economics, 00015 Monterotondo, Italy; valerio.faeti@crea.gov.it; 3 SSICA, Experimental Station for the Food Preserving Industry—Research Foundation, 43121 Parma, Italy; monica.bergamaschi@ssica.it (M.B.); cecilia.loffi@ssica.it (C.L.); anna.pinna@ssica.it (A.P.); roberta.virgili@ssica.it (R.V.)

**Keywords:** extensiveness, pig welfare, meat tenderness, fatty acids

## Abstract

Consumer demand for high-quality products, combined with expectations for more sustainable production systems and animal welfare, is driving major changes in livestock farming practices. It is known that space allowance plays a central role in pig welfare, promoting resting and reducing the incidence of injuries and stress-related behaviors; however, there is little scientific evidence on the effect that available space has on the carcass and meat quality. In this study, space allowances were compared, in both an indoor conventional system (1.15, 1.9 and 3 m^2^/pig) and an indoor organic system with outdoor access (1.4 + 1, 2.6 + 2 and 3.9 + 3 m^2^/pig). The increase in space available for pigs had no effect on pig performance, carcass and meat quality characteristics, such as pH, drip and cooking loss. However, lowering stocking density in the conventional indoor housing system improved meat tenderness, as assessed by the Slice Shear Force test, while no difference was found between meat tenderness in organic pigs raised with three different stocking densities. Increased space allowance per pig reduced n-3 fatty acids in pig loins from both housing systems and n-6 fatty acids and PUFAs in loins from pigs reared in the organic housing system with both indoor and outdoor space.

## 1. Introduction

Europe is considered one of the world’s leading suppliers of high-quality animal products, reinforcing consumers’ interest in product quality and origin and their confidence in European food systems [1]. Global demand for animal-based foods is expected to continue increasing in the coming decades, especially in developing countries [2], with pork representing the second largest source of animal protein after poultry, with a 34% share of global meat production in 2022, to which Europe contributes 24% [3]. Within the European context, Italian heavy pig production stands out for its economic importance and its association with high-quality, PDO-certified products. This system is characterized by extended fattening periods, higher slaughter weights and strict requirements concerning breed selection, feeding practices and animal welfare (AW) standards that ensure premium meat quality [4,5].

The growing consumer demand for high-quality products, coupled with expectations for more sustainable production systems, particularly regarding AW, is a highly relevant and timely issue in the livestock sector [6]. Extensive farming is characterized by lower resource use, a more sustainable land footprint and better AW than intensive farming, but may face challenges in consumer willingness to pay more [7,8]. AW, in particular, is a major challenge that most European pig producers have been facing in the last few decades and is associated with several aspects such as animal health, productivity and meat quality [9].

Among parameters affecting pig welfare, space allowance (SA) must be a guarantee, as it plays a central role, particularly in supporting natural behaviors such as resting and reducing the incidence of injuries and stress-related behaviors [10]. The EU Council Directive 2008/120/EC sets minimum SA for growing and fattening pigs according to the pig live weight [11]. For the heavy pig production, 1 m^2^/pig over 110 kg must be guaranteed.

Regulation (EU) 2018/848 [12] for organic farming asks for stocking density ensuring the animals’ welfare by providing them with sufficient space to stand naturally, to move, to lie down easily, to turn around, to groom themselves, to assume all natural postures and to make all natural movements; it repealed previous Regulation (EU) 889/2008 requiring 1.3 m^2^/pig indoor and 1 m^2^/pig outdoor spaces for fattening pigs in the 85–110 kg range without more space requirement for pigs with a live weight of over 110 kg [13]. Spoolder et al. [14] calculated the space requirement for pigs by using an equation that takes into account the body weight and the minimum physical space occupied by each pig.

A few studies explored the effect of different SAs on growth performance and carcass traits; Liorančas et al. [15] and Nannoni et al. [16] reported faster growth for the pigs in pens with extra space compared with those in smaller pens, likely due to easier access to feeders, but this trend was not observed in other studies [16]. Furthermore, a meta-analysis by Averos et al. [17] predicted different effects of SA depending on the presence/absence of a slatted floor on the feed conversion rate of growing finishing pigs. As for the effect of SA on meat quality, some studies compared conventional and organic farming systems, showing differences in meat quality, free amino acids and fatty acid composition [18,19].

Liorančas et al. [15] evaluated the effect of rearing space on pig behavior and meat quality, finding that 24 h pH was lower with less SA and that there was a tendency for pigs reared with a higher space at disposal to have a lower 24 h pH, a lower shear force, a lower a* index and a higher L*. Serrano et al. [20] reported a lower content of monounsaturated fatty acids (MUFAs) for pigs reared in larger space conditions. In the study of Nannoni et al. [16], α-linolenic acid (C18:3) was significantly higher in the experimental group reared in greater SA, while no differences were found in proximate composition pH_45′_, pH_24h_, colorimetric indices CIE L*, a* and b*, drip loss %, cooking loss %, and shear force. Similar results were reported by Lebret [21] for animals reared in a higher space allocation. Patton et al. [22] found a darker pork color score, as assessed by a trained sensory panel, and a lower polyunsaturated fatty acid (PUFA) content in lean tissue for pigs reared in a higher space allocation. However, it can be concluded that the impact of SA during rearing on meat quality remains a poorly investigated topic [23].

The present study specifically focuses on two rearing systems: a conventional indoor system and an organic system including both indoor and outdoor SAs, as required by organic farming regulations. The objective of the study was to investigate the effect of different SAs on the performance, carcass traits and meat quality of Italian heavy pigs, under controlled conditions where other influencing factors were kept constant.

## 2. Materials and Methods

This study encompassed two independent experiments taking place on two pig farms: the experimental farm owned and managed by the Italian Council for Agricultural Research and Analysis of Agricultural Economics, located in the municipality of San Cesario sul Panaro (MO), and a private organic pig farm located in the municipality of Fossano (CN). Both farms were selected according to their suitability and availability for hosting experimental trials with pigs and the high biosecurity standards that were implemented in Italy after the first ASF outbreak occurred in 2022. Ethical review and approval were waived for this study, which did not entail any harm to the observed pigs raised and kept under commercial conditions.

### 2.1. Animals

**Figure 1:**
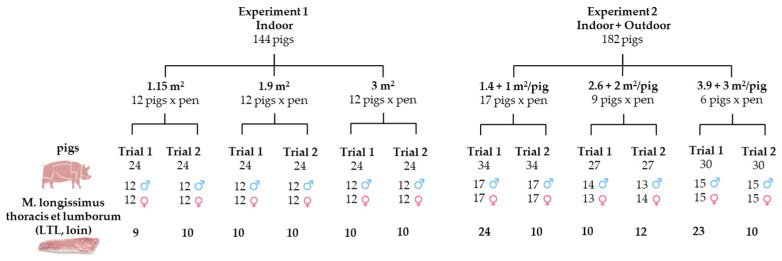
Experimental design showing the number of animals allocated to the different SA treatments and the number of loins sampled from each experimental group.

The experimental design is illustrated in Figure 1, which shows the number of animals in the different SA treatments. The figure also shows the number of loins collected for each experimental group.

#### 2.1.1. Pigs in Experiment 1

In total, 144 crossbred pigs (50%♀, 50%♂, Large White × Italian Duroc) with undocked tails were randomly assigned to three treatment groups housed indoors with three different SAs. The experiment was conducted over two consecutive trials. Each treatment group consisted of one pen of 12 female pigs and one pen of 12 barrows (i.e., castrated males). The experiment was then repeated. Each pig was identified by a tag or tattoo on the ear flap bearing a sequential identification number and was weighed (body weight, BW) during the experiment every two weeks by using a mobile electronic scale (DINI ARGEO, mod. DFWR, Fiorano Modenese, Italy). Daily feed intake was recorded per pen. Hot carcass weights also were recorded automatically on the slaughter line. Average daily gain (ADG) and feed conversion ratio (FCR) were calculated for the treatment groups.

#### 2.1.2. Pigs in Experiment 2

In total, 182 commercial hybrid pigs (50%♀, 50%♂, Topigs TN70 × Fomeva K-line) with undocked tails were randomly assigned and balanced by sex to three treatment groups housed in a pig house for organic production with three different SAs to have a balance in the sex of pen mates (50% barrows, 50% females). The experiment was conducted over two consecutive trials. Each pig was identified by a tag on the ear flap bearing a sequential identification number. Pigs in each treatment were weighed together at the beginning and end of each test session, due to the unavailability of an individual scale on the farm. Daily feed intake was recorded per pen. The growth performance of pigs in Experiment 2 was estimated based on the average live weight of all pigs in each group at the start and end of each trial, on the hot carcass weight of each pig, measured automatically on the slaughter line, and on the average dressing percentage of tested pigs.

### 2.2. Housing and Feeding

The SAs to be assigned to individual treatments were decided taking into account the limited scientific evidence available for trials in which carcass and meat quality were compared between pigs provided with standard stocking density or 30% [17] or 240% [16] more space [16]; it was therefore decided to test SAs well over (i.e., twice and three times) the standard minimum for conventional and organic farming [11,12].

Experiment 1 was carried out in an intensive indoor housing system with a fully solid floor and natural ventilation; pens were sized to keep 12 pigs at 1.15, 1.9 or 3 m^2^/pig. Each treatment group consisted of two pens, one for 12 females and one for 12 barrows, to ensure a sex balance between pigs within each treatment group. No bedding was used. Two metal chains hanging on the wall were provided in each pen as manipulable material. Pigs were fed twice a day (i.e., restricted) with flour milled feed distributed in a long trough (0.5 m/pig); one nipple drinker was available for every 12 pigs. Two types of compound feed were used for pigs in the growing and fattening phases: the first, for pigs weighing between 50 and 90 kg live weight, contained 14.39% crude protein (as-fed) and was composed of 25% corn, 30% barley, 21.5% sorghum, 10% wheat bran, 10% soybean, 0.46% synthetic amino acids and 3.04% vitamin and mineral supplements; the second feed, for pigs weighing between 90 and 180 kg live weight, contained 12.16% crude protein (as-fed) and was composed of 30% corn, 30% barley, 33.9% sorghum, 2.5% soya, 0.44% synthetic amino acids and 3.16% vitamin and mineral supplements. The average daily feed intake was 2.77 and 2.86 kg/day in the first and second trials, respectively.

Experiment 2 was conducted in a housing system for organic farming, naturally ventilated with a sloped straw-bedded solid floor inside and a concrete solid floor outside; a small portion of slatted floor was available inside the barn next to the drinkers and the door to access the outdoor run. Three different pig SAs were achieved by randomly assigning a different number of sex-balanced pigs to the pens: (1) two pens of 17 pigs per pen (34 pigs in total) with SAs of 1.36 m^2^/pig inside and 1.05 m^2^/pig outside; (2) three pens of 9 pigs per pen (27 pigs in total) with SAs of 2.57 m^2^/pig inside and 1.99 m^2^/pig outside; (3) five pens of 6 pigs per pen (30 pigs in total) with SAs of 3.85 m^2^/pig inside and 2.98 m^2^/pig outside. Pigs received liquid feed three times a day in a long trough (minimum 0.4 m/pig). Three types of compound feed for starters, growers and fatteners were used for all treatments, based on cereals and soybean with a vitamin and mineral supplement. Three different organic compound feeds were formulated for post-weaners, growers and fatteners, according to their growth stages. For pigs weighing between 30 and 80 kg, the feed contained 14.24% crude protein (as-fed) and was composed of 39.1% barley, 32.3% wheat, 3.2% wheat bran, 12.7% soybeans, 7.7% peas and 3.2% vitamin and mineral supplements. For pigs weighing between 80 and 120 kg, the feed contained 13.7% crude protein (as-fed) and was composed of 34.6% barley, 26.9% wheat, 8.6% wheat bran, 6.5% soybeans, 20.7% peas and 2.8% vitamin and mineral supplements. For pigs with a live weight of between 120 and 180 kg, the feed contained 12.65% crude protein (as-fed) and was composed of 25.4% barley, 34.4% wheat, 7.9% soybeans, 28.8% peas and 3.4% vitamin and mineral supplements. The average daily feed intake was 2.81 and 2.72 kg/day in the first and second trials, respectively. Feed dilution ratio was 1/2.6 (i.e., 2.6 L of water per kg of compound feed), and one functioning nipple drinker was also available in each pen for up to 17 pigs each.

The type and dosage of raw materials used in pig feed comply with the positive list and maximum quantities set by the Parma ham PDO scheme; pigs were restrictively fed in both experiments to meet the specifications for heavy pigs intended for Parma ham PDO production [24].

### 2.3. Pig Welfare Observation Before Slaughter

Pig welfare conditions were observed at the end of each experiment trial, the day before the pigs were loaded on a truck before being transported to the slaughterhouse, by taking resource- and animal-based measures to assess AW, as recommended by EFSA [25]. Resource-based measures were used to assess the degree of litter coverage and the level of soiling in pens, while animal-based measures were used to assess pig behavior (e.g., avoidance, manipulation), dirtiness, lesions (tail, body wounds, hernia, lameness) and mortality. The data collected were not processed statistically because these few observations were not repeated as they were only aimed at obtaining information about AW at the end of the experiments.

The presence of straw or other bedding in pens/resting areas was observed and scored according to following modified definitions given by the SusPigSys project [26]: whole area is bedded, score 1; at least lying area with bedding, but not whole pen, score 2; not all pigs can lie on bedded area, score 3; no bedding, score 4. The SusPigSys protocol [26] was also used to assess the level of dirt in each pen and pig manipulation behavior. Pen dirtiness was scored as follows: clean (i.e., not more than 10% of the area is wet or damp or dirty with feces)—score 1; medium dirtiness—score 2; dirty (i.e., >50% of the area is wet or damp or dirty with feces)—score 3. Pig manipulation behavior was measured in terms of percentage of observed active pigs manipulating different pen elements (i.e., wall, floor, pen fixture, chain) or other pen mates.

Pig fear of humans was assessed, as avoidance behavior, by checking the percentage of pigs showing panic/avoidance of approach by an unknown assessor entering the pen and standing there for 30 s and then walking slowly around the pen for a further 30 s. The number of pigs showing panic/avoidance, defined as fleeing or facing away from the assessor, including huddling in the corner, but not including play-like avoidance, is reported as a percentage of the number of visible pigs, according to the modified Welfare Quality^®^ protocol for assessing pig welfare [27].

Pigs were also clinically inspected for detecting short or stump tails, moderately or severely dirty pigs and lameness, according to SusPigSys [26], and body wounds and hernia, according to Welfare Quality^®^ protocol [27], in terms of % of affected pigs. The mortality rate for each treatment was reported, including pigs dropped from the experiments for health reasons, as determined by the farm vet’s assessment.

### 2.4. Climate Conditions

The pigs tested in each trial of the two experiments were raised indoors in the same room with access, for organic pigs, to outdoor pens to ensure the same microclimatic conditions. The outside air temperature and relative humidity provide information about the local climate, which influences the microclimatic conditions outside and also inside the sheds used in Experiments 1 and 2, as they were naturally ventilated without any cooling treatment of the incoming air. The minimum, maximum and average daily temperatures and average relative humidity in the municipalities of San Cesario sul Panaro (MO) and Fossano (CN) [28] were analyzed with reference to the duration of the experiments.

Experiment 1: The outside air temperature in the geographic area where the farm is located was 12.4 °C on average, with variations from −4.9 to 35.5 °C, whereas the mean relative humidity was 75.4% during the first trial of Experiment 1, from 31 December 2022 to 4 July 2023. During the replica, from 7 August 2023 to 8 January 2024, the air mean temperature was 13.3 °C, varying from −4.8 to 38.3 °C, while the mean relative humidity was 72.5%.

Experiment 2: The outside air temperature was 19.9 °C on average, ranging from 6.1 to 35.6 °C, while the mean relative humidity was 72.5%, in the first trial from 4 May 2023 to 17 October 2023. During the replica, from 5 December 2023 to 19 May 2024, the outside air mean temperature was 6.9 °C, ranging from −10.6 to 26.2 °C, and mean relative humidity was 79.2%.

### 2.5. Carcass Measurement and Loin Sampling

The hot processing of heavy Italian pig carcasses, commonly used in the production of cured meats, involves several stages that transform the carcass into finished products. These stages include slaughtering, weighing the carcass and cutting into different primal cuts according to the Italian method [29], followed by cooling. Pig carcasses were assessed, and the left longissimus thoracis et lumborum (L. dorsi, loin) was collected on a subset of pigs involved in the experiments; the experimental design involved sampling at least ten loins at the end of each trial from each of the three treatment groups, five from female pigs and five from barrows, selected at random (Figure 1).

The loins were sampled and chilled on the day of slaughter and delivered the following day to the SSICA laboratories.

In total, 59 out of the 144 pigs involved in Experiment 1 and 89 out of the 182 pigs involved in Experiment 2 had their carcasses assessed and their left loins sampled. The sampled loins were chilled on the day of slaughter and delivered to the SSICA laboratories the following day.

In the first experimental session of Experiment 2, the slaughterhouse and additional intrinsic data on the meat (i.e., on left loins) were collected for 48 pigs.

The classification of pig carcasses was carried out in accordance with Decision 2014/38/EU [30]. A Fat-O-Meat’er II (FOM II) probe was used to measure backfat thickness (including skin) (mm), muscle thickness (mm) and lean meat (%).

**Figure 2:**
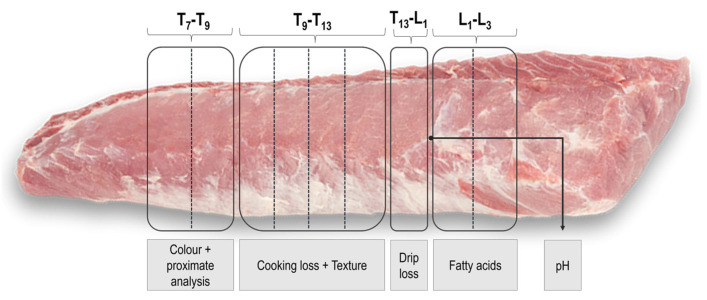
Sampling procedure for analyses including slices taken from T7 thoracic vertebra to L3 lumbar vertebra of the longissimus thoracis et lumborum.

At 48 h postmortem, the pH was measured at the first lumbar vertebrae (L1) position. Subsequently, four subsamples were collected from the loin according to the scheme shown in Figure 2, and each section was designated for a specific analysis. Specifically, the seventh and ninth thoracic vertebrae (T7–T9) section was for color and proximate composition analysis; T9–T13, for cooking loss and texture analysis; T13–L1, for drip loss analysis; and L1–L3, for fatty acid analysis.

Subsamples for color and drip loss were analyzed immediately. Fatty acid sections were divided into two slices, each wrapped separately in aluminum foil, vacuum-packed, and frozen at −20 °C. Subsamples for cooking loss and texture analysis were wrapped in plastic to prevent dehydration and then stored at 4 °C to mature until 72 h postmortem. At this point, they were cut into four slices, each 3.5 cm thick, vacuum-packed, and frozen at −20 °C for subsequent analyses.

### 2.6. Meat Quality Analyses

#### 2.6.1. pH at 24 and 48 h

The pH was measured at 24 and 48 h postmortem in the loin muscle and determined using a pH meter (Portavo 920, Knick, Berlin, Germany) equipped with a penetration probe electrode (XS sensor) and a temperature probe for automatic temperature compensation. Three pH values were recorded for each sample in different locations at the level of the L1 vertebra.

#### 2.6.2. Color

Color measurement was performed using a Konica Minolta CM-700d Spectrophotometer (Osaka, Japan) with the following settings: illuminant D65, 10° standard observer, 8 mm aperture, and specular component excluded. The instrument was calibrated against a white plate (CM-A177) provided by the manufacturer, while pink and red plates (Konika Minolta, Tokyo, Japan) were used as reference targets prior to initiating sample measurements. Two slices, each 3 cm thick, were obtained from the T7–T9 loin subsamples and allowed to rest for blooming (30 min at 4 °C). Five measurements were taken at different positions on each slice. The CIE parameters lightness (L*), redness (a*) and yellowness (b*) were determined, and the hue angle (h◦) and chroma (C*) indices were calculated. The average of the readings was computed for each sample. Subsequently, all samples were homogenized and stored at −20 °C for further analysis.

#### 2.6.3. Drip Loss

Drip loss was measured 48 h postmortem following the method described by Rasmussen & Andersson [31]. A 20 mm slice taken at the level of the T13–L1 was cut perpendicular to the muscle fiber direction. Within a few seconds, two samples were cored along the fiber direction from the center of the meat slice using a Ø 25 mm core borer. The samples were then placed in a specialized plastic container and stored at 4 °C for 24 h prior to weighing and calculation.

#### 2.6.4. Proximate Composition

Proximate composition was determined according to AOAC methods [32] in the homogenized T7–T9 subsample following color analysis. Moisture (%) was determined gravimetrically by drying about 2 g of sample in an oven at 105 °C until constant weight (AOAC 950.46). Fat content (%) was measured using Soxhlet extraction with petroleum ether as described in the AOAC 991.36 method. Total nitrogen (%) was determined by the Kjeldahl method (AOAC 981.10), and the results were expressed as protein (%) using a conversion factor of 6.25.

#### 2.6.5. Texture Analysis (Slice Shear Force) and Cooking Loss

Cooking loss and texture analyses were performed on two 3.5 cm thick slices cut from the T9-T13 section after 72 h of maturation at 4 °C. Prior to analysis, the samples were thawed at 4 °C for 24 h and weighed. The slices were grilled at 200 °C on a preheated plate grill (Royal Catering, Singapore), with internal temperature monitored using a digital Thermometer (PCA Italia s.r.l., mod. T390, Capannori, Italy) equipped with four thermocouples. Upon reaching an internal temperature of 40 °C, the sample was flipped and grilled until 68 °C and then allowed to rest until the temperature stabilized at 70 °C. The final weight after cooking was recorded to calculate cooking loss. The Slice Shear Force (SSF) method was applied to two slices of pork meat according to the procedure described by Pinna et al. [33]. A 5 cm portion was cut from each slice; then, two parallel cuts, 1 cm apart, were made along the length of the portion, at a 45-degree angle to the loin axis, parallel to the muscle fibers. Two specimens were obtained from each slice.

The specimens were sheared using a flat, blunt-point blade with a thickness of 1.1684 mm and a half-round beveled cutting edge, attached to a Universal Testing Machine (INSTRON, mod. 5565, IL, USA)) equipped with a 500 N load cell. The test was performed with a crosshead speed of 500 mm/min. The measured parameters included shear force (maximum shear force, in N) and fracturability (N).

#### 2.6.6. Fatty Acid Analyses

Fatty acid analysis was performed on a 1.5 cm thick slice cut from the L1–L3 section at 48 h after slaughter and stored at −20 °C for up to 3 months. Total lipid extraction was carried out following the method of Folch et al. [34], while acid-catalyzed transmethylation was performed according to the UNI EN ISO 12966-2:2017 method [35] with some modifications, using PESTINORM^®^-grade solvents and anhydrous glassware. Thirty grams of homogenized loin meat trimmed of subcutaneous fat were homogenized with 100 mL of chloroform/methanol (2:1) containing 0.01% butylated hydroxytoluene and filtered through 80 g/m^2^, 43–48 µm paper; 35 mL of distilled water and a spatula of graphitized carbon were added to the meat suspension, which was then centrifuged at 15,000× *g* for 15 min at 4 °C. The organic phase was recovered by filtration through 0.22 µm hydrophobic filters (GVHP, Millipore, Burlington, MA, USA) and allowed to rest for 2 h with a spatula of anhydrous sodium sulfate before drying in a rotary evaporator at 40 °C to recover the fat. Transmethylation was performed in a boiling water bath (100 °C, 1 h); 100 mg of extracted fat (two replicates for each sample) was dispersed in 800 µL of a 15:1 MeOH/H_2_SO_4_ solution, and 100 µL of a 50 mg/mL hexane solution of C19:0 was added as internal standard for FAMEs. After cooling, 2 mL of distilled water and 1 mL of hexane were added, mixed and centrifuged (3750× *g*, 10 min, 4 °C). The resulting upper organic layer was filtered and transferred into amber vials.

GC analysis of FAMEs was performed employing a GC-FID system (Trace GC Ultra, Thermo Fisher Scientific, Waltham, MA, USA) equipped with an SP 2380 GC capillary column (100 m × 0.25 mm × 0.2 µm). The oven temperature program was as follows: hold at 100 °C for 2 min, increase to 250 °C at a rate of 4.5 °C/min and hold for 15 min. The carrier gas was helium at a flow rate of 1.3 mL/min. The identification of each individual FAME was performed using an external quantitative standard mixture (FAME Mix 37 components C4–C24, (Supelco Inc., Bellefonte, PA, USA)) added with methyl all cis-7,10,13, 16-docosatetraenoate (C22:4) and methyl all cis-7,10,13, 16, 19-docosapentaenoate (C22:5) analytical standards (Supelco Inc., Bellefont, PA, USA). The response factors were calculated by using the methyl nonadecanoate (C19:0) analytical standard (Supelco Inc., Bellefont, PA, USA). Quantification was performed according to the method described in UNI EN ISO 12966-4:2015 [34]. Results were expressed as fatty acid percentage of total fatty acid content (% of total FAs), presented as the mean of the two transmethylation replicates.

### 2.7. Statistical Analysis

Statistical analysis was performed by using the General Linear Model (GLM) procedure with the application of one-way ANOVA. The regression analysis in the GLM was used to verify the effect of slaughter weight on the meat and carcass parameters and whether additional corrections should be made. The model included the SA conditions and the sex of animals as fixed effects and the trial replica as a random effect (sex and replica effects were not reported in tables). The Bonferroni post hoc test was applied to compare the estimated marginal mean values (EMMs) for significant differences (*p* < 0.05). All statistical analyses were performed with IBM SPSS statistics ver. 29 (SPSS Inc., Chicago, IL, USA). Normal distribution of data was inspected before statistical analysis.

## 3. Results

### 3.1. Growth Performance and Carcass Quality

The results of initial and final body weights, average daily gain (ADG) and feed conversion ratio (FCR) from both experiments are summarized in Table 1 according to the different SAs.

Three pigs in the intermediate treatment group (i.e., 1.9 m^2^/pig) dropped from the experiment at an early stage due to health issues, namely death, severe lameness and abscess, and were replaced by three additional pigs to maintain the intended stocking density in the treatment group. In both experiments, pigs were slaughtered at high live weights: approximately 190 kg in Experiment 1 and 170 kg in Experiment 2.

The analysis of variance revealed no significant differences in ADG and FCR among the SAs considered in the two experiments. Similarly, when considering the sex factor, no difference was found between the different treatments, which could be due to the feed restriction needed to comply with the Parma ham PDO production specifications. Carcass quality data of backfat thickness, muscle thickness and lean meat percentage are given in Table 2.

The analysis of variance revealed no significant differences in carcass parameters (backfat thickness, muscle thickness, lean meat) among SAs considered in the two experiments and therefore no effect on these parameters due to different levels of SA in the two housing systems.

### 3.2. Pig Welfare Observation Before Slaughter

The outputs of AW observations at the end of each of the two experiments were focused on bedding coverage and pen dirtiness, pig avoidance and manipulation behaviors, clinical observations and mortality rate (Table 3 and Table 4).

### 3.3. Meat Quality Parameters

The results of the meat quality parameter analyses obtained from Experiments 1 and 2 are shown in Table 5 and Table 6, respectively.

In Experiment 1, thawing loss was higher in samples from pigs reared in the restricted pen compared to those in the intermediate SA.

No significant differences were observed in most of the meat quality parameters among pigs reared under different SA conditions in both experiments (pH at 24 h and 48 h postmortem, drip loss, cooking loss and some CIE L* a* b* colorimetric indices). In Experiment 1, thawing loss was higher in samples from pigs reared in the restricted pen compared to those in the intermediate SA.

In Experiment 2, samples from the intermediate SA group exhibited a higher L* (lightness) compared to the restricted pen group (*p* < 0.05), accompanied by a lower a* (redness) (*p* < 0.05).

Results from Experiment 1 showed a significant reduction in shear force (*p* < 0.05) and fracturability (*p* < 0.05) in loins obtained from pigs with increased space availability.

In contrast, no significant differences in texture parameters were observed in Experiment 2, resulting in extremely low shear force values, indicating a good level of tenderness across treatments [33].

In both experiments, loin color differences were associated with the sex of the pigs: barrows have a higher redness index (a*) (2.41 vs. 1.55 in Experiment 1 and 2.28 vs. 1.53 in Experiment 2) and lower red shade (hue angle) than females (72.2 vs. 83.0 in Experiment 1 and 80.3 vs. 83.5 in Experiment 2). In Experiment 2, differences between barrows and gilts were also found in chroma (C*) (13.0 vs. 12.5), moisture content (72.4% vs. 73.2%), and intramuscular fat content (4.31% vs. 3.50%).

### 3.4. Effects of SA on Fatty Acid Composition in the Rearing Conditions of Experiments 1 and 2

Fatty acid analysis was performed in order to evaluate the effect of the housing conditions from a nutritional perspective. Table 7 reports the results from Experiment 1, whereas Table 8 shows the results from Experiment 2.

In Experiment 1, pigs reared with the highest indoor SA (3 m^2^) showed lower levels of single omega-3 fatty acids (C18:3 n-3 ALA, C22:6 n-3 DHA) as well as the sum of omega-3 fatty acids, while the n-6/n-3 ratio was significantly higher. Omega-3 fatty acids and the n-6/n-3 ratio largely rely on factors like feeding and genetics [36]. As for single fatty acids, significant differences were observed in SFAs C10:0, C12:0 and C22:0, which were lower in the pigs reared in the highest SA, and in monounsaturated fatty acids C14:1, C18:1 trans-9 and C22:1 cis-9, which were significantly lower with increasing SA.

In Experiment 2, several differences in the fatty acid profile were reported in relation to different SAs.

The total MUFA content was higher in samples from pigs provided with the highest SA, while the total PUFA and n-3 fatty acid levels were correspondingly lower in this group. Single MUFAs C18:1 n-9 cis and C18:2 n-6 cis and single n-3 fatty acids ALA, EPA, C22:5 n-3 and DHA gave a relevant contribution to differences in the sum of MUFAs, PUFAs and n-3 fatty acids.

## 4. Discussion

In the present study, two experiments were performed, following two distinct farming systems: a conventional indoor production system (Experiment 1) and an organic system including both indoor and outdoor areas (Experiment 2). Animal genetic lines, feeding regimens, and overall management differed between the two experiments as they are specific to each production system and context. Therefore, the aim of this study is to assess the effects of SA on pig growth performance, carcass traits, and meat quality parameters within two different production systems.

No statistically significant differences were found in growth performance and carcass quality between the groups of pigs treated in both experiments, which could be partly attributed to the feed restriction imposed on heavy pigs to achieve the quality target of the Parma ham PDO specification. These results are consistent with a study on lean pigs raised in hoop structures on a cornstalk-straw-bedded floor and fed ad libitum [22] but in contrast to two other studies on lean pigs raised on a slatted floor and fed ad libitum [15] and on heavy pigs kept on a slatted floor and subjected to restricted feeding [16]. These findings suggest that growth and carcass quality may be influenced by factors beyond SA, including the feeding system, pig competition, type of floor and slaughter weight. However, these previous studies all refer to a limited increase in the area of housing, while the present study compares SA levels up to three times the minimum indicated by the regulations for conventional and organic pig farming.

Pig welfare observation showed reduced pen dirtiness and lower percentages of moderately and severely dirty pigs in pens with higher SA. The relatively high level of dirtiness in the pens and pigs in Experiment 2 may be influenced by liquid feeding, which was shown to cause more soiling than wet and dry feeding systems [37].

The higher percentage of pigs exhibiting panic/avoidance behavior towards unfamiliar humans in larger pens can be explained by the greater space available and therefore the greater opportunity for pigs to move away from the person taking the measurement. Manipulation behaviors towards the floor and pen fixtures (i.e., metal partitions) were observed to a greater extent in pens with less SA. The lower incidence of pigs manipulating the floor, pen fixtures and chain observed in Experiment 2 can be attributed to the pigs’ reduced interest in these environmental elements, due to the greater sensory stimuli provided by the accessible outdoor area and the weekly distribution of straw (150 g/day*pig), which also serves temporarily as manipulable material before being soiled by pig feces and urine.

Outputs of pig clinical observation and mortality rates show that short and docked tails were observed only in Experiment 1, in which pigs were raised on solid flooring without any type of bedding, while in Experiment 2, pigs were provided with straw as bedding on a weekly basis, which can also serve as manipulable material to prevent tail biting [38]. However, no fresh tail lesions were clinically observed, suggesting that no tail biting occurred in the final stage of the fattening period.

In Experiment 1, where pigs were reared entirely indoors, no significant differences were observed in key meat quality parameters such as pH, drip loss, proximate composition and cooking loss. In this context, the variation in SA did not appear to influence these quality traits, which are known to be more strongly affected by other factors such as pre-slaughter management, dietary composition and genetic background [23]. These results align with previous studies reporting limited or inconsistent effects of SA on meat quality traits [16,20,22,23], suggesting that, under controlled indoor conditions with restricted feed regimes, space alone may have a limited effect on some intrinsic meat quality parameters.

In contrast, an impact of SA was detected on thawing loss and meat texture, particularly in Experiment 1. As space availability increased, a significant reduction in Slice Shear Force (maximum shear force and fracturability) indicated an improved tenderness. The reduction in shear force can be partially explained by lower thaw loss, resulting in improved moisture retention and thus greater tenderness. Conversely, no significant differences were detected in Experiment 2, as loins from all SA groups were characterized by high tenderness [32]. It is worth noting that even pigs in the group with the lowest SA in Experiment 2 were kept under conditions comparable to those with the highest SA in Experiment 1. This result may also be influenced by the different conditions of Experiment 2 in terms of genetics, feeding, animal management, content and quality of fat, such as the higher contribution of IMF and PUFAs. Texture is a meat quality parameter that has been rarely investigated in relation to SA [15,16,22,35]. Liorančas et al. [15] reported a reduction in shear force when space allowance was more than doubled (0.5 m^2^/pig to 1.2 m^2^/pig), and Jang et al. [35] found that shear force linearly increased as floor space decreased. In spite of this, many authors did not detect differences in the shear force of loins attributable to SA [23], even if, in general, small differences in SA were assayed: Nannoni et al. [16] compared 1.0 m^2^/pig to 1.3 m^2^/pig, and Patton et al. [22] compared 0.70 m^2^/pig to 1.13 m^2^/pig. The small spatial variations tested in those trials may not have been sufficient to influence muscle structure or postmortem proteolysis responsible for the development of tenderness.

This finding suggests that pigs raised with more space may experience less stress, thus positively influencing tenderness assayed at 72 h postmortem [15,36]. The biological basis for these differences may be related to protein turnover dynamics and to postmortem proteolysis of cytoskeletal and myofibrillar proteins, processes crucial for tenderization that are strongly influenced by the oxidative status of the muscle [39].

Goldberg et al. [40] showed that the rate of protein degradation depends upon the level of muscular activity, while Sarri et al. [41] report that stimuli like physical activity, together with increased nutrient availability in the diet (e.g., amino acids), allow for more efficient protein synthesis. The balance between protein synthesis and degradation defines protein turnover, affecting tissue deposition. Rennie et al. [42] reported that physical exercise by adult humans increased the rate of protein breakdown and decreased protein synthesis.

In our study, the feeding type and level were the same in the pig groups differing in SA; under the experimental conditions adopted, the group of pigs with the largest availability of space (3.9 + 3 m^2^/pig in Experiment 2) showed a tendency, though not significant, to yield the lowest slaughter weight. If this condition implies a prevalence of protein degradation in the protein turnover balance, this supports the relationship with tenderness of groups with greater space availability.

Fatty acids are an important meat quality indicator, also from a nutritional perspective [18]. Fatty acid profile is highly influenced by various rearing parameters, like conventional/organic farming and diet, to a large extent [21]. In this work, we focused our attention on the effect of the housing system, while keeping other rearing parameters, like diet, fixed.

In Experiment 1, no significant differences were found in the loins of pigs reared indoors for the different classes of fatty acids as SA increased, with the exception of the n-6/n-3 ratio, which was significantly higher in pigs reared with the highest SA (3 m^2^).

Nannoni et al. [16] reported no significant differences in the fatty acid profile of subcutaneous fat of green hams from heavy pigs raised at different floor SAs, with the exception of α-linolenic acid (C18:3 n-3 ALA), which had higher values in pigs with individual SAs. A higher value for this fatty acid, however, has already been reported in other studies for animals reared in a higher SA, especially if access to an outdoor area with free grazing is at disposal [21], as a consequence of diets richer in omega-3 fatty acids. In the present study, the feeding regimen was consistent across pig groups within each experiment.

In Experiment 2, a decreasing PUFA level was observed with increasing SA, involving both n-3 fatty acids (C20:5 n-3 EPA, C22:5 n-3, C22:6 n-3 DHA, sum of n-3 fatty acids) and n-6 fatty acids (C18:2 n-6 cis, C20:2 n-6, C20:4 n-6, sum of n-6 fatty acids) as reported for pigs reared in outdoor conditions. The reduction in PUFA content can be attributed to increased physical activity for animals reared with more SA, as previously reported as a metabolic consequence of exercise training [43,44]. The fatty acid profile pattern of pigs with the highest level of SA was consistent with the pattern found in pigs trained in physical exercise [45,46]. One possible explanation for the observed reduction in PUFA levels could lie in an alteration in fatty acid metabolism and an increase in fatty acid oxidation during exercise [47,48]. However, it should be noted that pigs from Experiment 2 showed higher intramuscular fat content than pigs in Experiment 1; therefore, considering the fatty acid profile alone, a reduction in PUFAs, which are mainly found in membrane phospholipids, may be also result from an increase in other fatty acid classes, such as SFAs and MUFAs, which are mainly present in neutral lipids. In contrast, other studies reported a higher total PUFA level in the intramuscular neutral fat for exercised pigs compared to sedentary ones [49]. A different fatty acid composition can therefore result from physical training, which influences the composition of muscle fiber types and, in turn, affects the fatty acid profile [50]. A modified fatty acid metabolism as a consequence of physical exercise could partly explain the fatty acid profile of Experiment 1, even though the reduction in PUFAs was mainly limited to omega-3 fatty acids in the pig group provided with the highest SA. In Experiment 2, a higher level of MUFAs was observed for a greater space available. In this same experiment, a significantly higher level of oleic acid (C18:1n-9) was reported for the largest SA, as reported by other authors as a consequence of increased physical activity [44,51]. Patton et al. [22] reported similar results for gilts reared in deep-bedded hoop structures with an increasing degree of SA; for increased space allocation, higher but not significantly higher levels of SFAs and MUFAs were observed, along with lower levels of PUFAs and the n-6/n-3 ratio. From a nutritional point of view, a lower n-6/n-3 ratio is desirable, with n-6/n-3 close to 4 being the optimum value [52]. In the present study, an n-6/n-3 ratio close to 7 was achieved, as already reported for pigs raised both indoors and outdoors [53].

The higher variability in fatty acid composition observed in Experiment 2 compared to Experiment 1 may result from several key factors: differences in husbandry conditions (including genetic background and feeding regimes) and the increased physical activity allowed by partial outdoor access. These factors can modify fat content and its distribution in lean tissue, ultimately influencing the profile and variability of fatty acids.

## 5. Conclusions

In conclusion, the increase in SA did not result in significant changes in growth performance or carcass characteristics in Experiment 1 (conventional production system) and in Experiment 2 (organic production system), as both were managed to achieve standardized growth rates in accordance with the strict specifications required for the production of PDO Parma ham.

An improvement in meat tenderness was observed in the conventional indoor system at higher SAs, in terms of lower shear force and fracturability values. The same effect was not highlighted in the organic system with indoor and outdoor spaces, possibly due to the high SA available to all groups. Other parameters, such as pH, drip loss and proximate composition, were not affected by SA. As regards changes in fatty acid profile, it appears that increased physical exercise causes a reduction in PUFAs due to oxidation, which was limited to omega-3 fatty acids in the case of the indoor experiment and extended to n-6 fatty acids in the indoor and outdoor experiment characterized by a greater physical activity of pigs.

Further research is needed to investigate the effect of higher SAs, such as in outdoor free-range and extensive farming systems, on pig growth, carcass traits and meat quality, under controlled conditions.

## Figures and Tables

**Table 1:** Estimated marginal mean (EMM) and standard error of mean (SEM) of carcass performance parameters: initial and final body weight (BW), average daily gain (ADG) and feed conversion ratio (FCR) of conventional heavy pigs reared indoors (Experiment 1) and organic heavy pigs reared indoors and outdoors (Experiment 2) with different levels of SA.

Space Allowance										
	Experiment 1—Indoor					Experiment 2—Indoor + Outdoor				
	1.15 m^2^/pig	1.9 m^2^/pig	3 m^2^/pig			1.4 + 1 m^2^/pig	2.6 + 2 m^2^/pig	3.9 + 3 m^2^/pig		
Pigs (*n*.)	48	45	48			34	22	33		
	EEM	EEM	EEM	SEM	*p*	EEM	EEM	EEM	SEM	*p*
Bw_initial_ (Kg)	52.06	52.39	52.14	0.60	*0.922*	37.3	36.9	31.1	*N.A ^1^*	*N.A ^1^*
Bw_final_ (Kg)	190.7	191.4	189.9	1.99	*0.871*	178.0	170.3	170.8	3.56	*0.265*
ADG (Kg/d)	0.749	0.754	0.752	0.009	*0.945*	0.836	0.799	0.832	0.022	*0.506*
FCR	3.786	3.790	3.783	0.045	*0.995*	3.621	3.743	3.602	0.093	*0.547*

^1^ N.A: not applicable. The GLM statistical analysis was not performed for Bw_initial_ in Experiment 2 because only group means were available (not individual animal data); therefore, the weighted mean of Experiment 2 is reported.

**Table 2:** EMM and SEM of carcass quality parameters of conventional heavy pigs reared indoors under different conditions of SA (Experiment 1) and of organic pigs reared indoors and outdoors under different conditions of SA (Experiment 2).

Space Allowance										
	Experiment 1—Indoor					Experiment 2—Indoor + Outdoor				
	1.15 m^2^/pig	1.9 m^2^/pig	3 m^2^/pig			1.4 + 1 m^2^/pig	2.6 + 2 m^2^/pig	3.9 + 3 m^2^/pig		
Pigs (*n*.)	48	45	48			34	22	33		
	EEM	EEM	EEM	SEM	*p*	EEM	EEM	EEM	SEM	*p*
Backfat thickness (mm)	29.63	29.09	29.91	0.67	*0.684*	28.51	28.16	26.64	1.45	*0.566*
Muscle thickness (mm)	69.18	68.06	65.83	1.31	*0.183*	66.27	61.50	63.49	1.99	*0.329*
Lean meat (%)	52.35	52.56	52.13	0.31	*0.625*	52.73	53.27	53.58	0.69	*0.637*

**Table 3:** Bedding coverage and pen dirtiness in the resting area (scores) and pig avoidance and manipulation behaviors (%). Median values of scores and mean percentages of pigs for the combined two replicates per experiment.

Space Allowance						
	Experiment 1—Indoor			Experiment 2—Indoor + Outdoor		
	1.15 m^2^/pig	1.9 m^2^/pig	3 m^2^/pig	1.4 + 1 m^2^/pig	2.6 + 2 m^2^/pig	3.9 + 3 m^2^/pig
Bedding in rest area (score 1 to 4) ^1^	4	4	4	2	2	2
Dirtiness in rest area (score 1 to 3) ^2^	2	1.5	1	3	2	2
Avoidance behavior (% of visible pigs)	10	17	27	12	63	98
Manipulation behavior (% of active pigs)						
Wall	0	2	0	2	4	2
Floor	17	26	16	7	4	7
Pen fixture	33	24	18	5	5	2
Chain	8	6	0	0	0	0
Pig	2	0	5	12	6	2

^1^ from 1 (whole area covered) to 4 (no litter); ^2^ from 1 (clean) to 3 (dirty).

**Table 4:** Clinical observation of pigs prior to slaughter and mortality rate. Average percentages of pigs for the combined two replicates per experiment.

Space Allowance						
	Experiment 1—Indoor			Experiment 2—Indoor + Outdoor		
	1.15 m^2^/pig	1.9 m^2^/pig	3 m^2^/pig	1.4 + 1 m^2^/pig	2.6 + 2 m^2^/pig	3.9 + 3 m^2^/pig
Short tail	2	0	10	0	0	0
Stump tail	0	0	4	0	0	0
Moderately dirty	52	25	17	64	48	39
Severely dirty	19	0	0	30	15	15
Mild body wounds	0	4	0	7	0	0
Severely lame	2	0	0	4	5	0
Hernia	2	0	0	0	0	3
Mortality rate	0	8	0	0	6	2

**Table 5:** EMM and SEM of meat quality parameters assayed in loins of pigs reared indoors under different conditions of SA (Experiment 1). Within a row, different letters denote significant differences among sired lines.

Space Allowance in Experiment 1—Indoor					
	1.15 m^2^/pig	1.9 m^2^/pig	3 m^2^/pig		
Samples (*n*.)	19	20	20		
	EMM	EMM	EMM	SEM	*p*
pH_24h_	5.64	5.62	5.62	0.02	*0.690*
pH_48h_	5.64	5.64	5.63	0.02	*0.755*
Drip loss	1.58	1.51	2.21	0.22	*0.413*
Thawing loss	6.39 ^a^	5.14 ^b^	5.77 ^a,b^	0.24	*0.002*
Lightness (L*)	52.3	50.9	51.6	0.61	*0.276*
Redness (a*)	1.99	2.09	1.87	0.25	*0.821*
Yellowness (b*)	11.3	11.0	11.1	0.23	*0.769*
Chroma	11.6	11.2	11.4	0.26	*0.455*
Hue angle (h°)	80.9	80.6	81.8	1.14	*0.746*
Moisture (%)	73.3	73.0	73.3	0.15	*0.367*
Protein (%)	22.8	23.1	23.1	0.14	*0.070*
Fat (%)	3.31	2.94	2.83	0.22	*0.270*
Cooking loss (%)	18.6	18.2	19.5	0.66	*0.386*
Slice Shear Force (N)	111.9 ^a^	101.6 ^a,b^	95.1 ^b^	3.7	*0.046*
Fracturability (N)	22.7 ^a^	18.5 ^a,b^	16.0 ^b^	0.9	*0.017*

**Table 6:** EMM and SEM of meat quality parameters assayed in loins of pigs reared indoors and outdoors under different conditions of SA (Experiment 2). Within a row, different letters denote significant differences among sired lines.

Space Allowance in Experiment 2—Indoor + Outdoor					
	1.4 + 1 m^2^/pig	2.6 + 2 m^2^/pig	3.9 + 3 m^2^/pig		
Samples (*n*.)	34	22	33		
	EMM	EMM	EMM	SEM	*p*
pH_24h_	5.60	5.62	5.57	0.015	*0.071*
pH_48h_	5.57	5.61	5.58	0.014	*0.158*
Drip loss	1.78	1.42	1.73	0.215	*0.511*
Thawing loss	6.015	6.293	6.540	0.325	*0.330*
Lightness (L*)	55.3 ^a^	57.2 ^b^	55.8 ^a,b^	0.574	*0.037*
Redness (a*)	2.34 ^a^	1.42 ^b^	1.95 ^a,b^	0.871	*0.042*
Yellowness (b*)	12.6	12.5	12.3	0.194	*0.294*
Chroma	13.0	12.7	12.6	0.224	*0.267*
Hue angle (h°)	80.2 ^b^	84.0 ^a^	81.6 ^a,b^	1.212	*0.042*
Moisture (%)	73.2 ^a^	72.8 ^a,b^	72.3 ^b^	0.235	*0.001*
Protein (%)	22.8	22.7	22.6	0.125	*0.367*
Fat (%)	3.54	3.92	4.26	0.218	*0.073*
Cooking loss (%)	20.6	20.6	20.7	0.654	*0.994*
Slice Shear Force (N)	82.4	73.6	79.2	2.871	*0.094*
Fracturability (N)	14.5	12.4	13.3	2.589	*0.203*

**Table 7:** EMM and SEM of fatty acids (FAs) as % of total FAs assayed in loins of pigs reared indoors under different conditions of SA (Experiment 1). Within a row, different letters denote significant differences among sired lines.

	Space Allowance in Experiment 1—Indoor				
	1.15 m^2^/pig	1.9 m^2^/pig	3 m^2^/pig		
Samples (*n*.)	19	20	20		
	EMM	EMM	EMM	SEM	*p*
FAs (% total FAs)					
C10:0	0.15 ^a,b^	0.16 ^a^	0.13 ^b^	0.004	*0.001*
C12:0	0.11 ^a^	0.11 ^a^	0.09 ^b^	0.006	*0.049*
C13:0	0.07 ^b^	0.13 ^a^	0.07 ^b^	0.02	*0.013*
C14:0	1.47	1.46	1.38	0.04	*0.142*
C14:1 *cis-9*	0.07 ^a,b^	0.09 ^a^	0.04 ^b^	0.01	*0.001*
C15:0	0.06	0.07	0.14	0.06	*0.238*
C15:1 *cis-10*	0.05	0.06	0.043	0.01	*0.423*
C16:0	23.12	23.37	23.34	0.21	*0.380*
C16:1 *cis-9*	3.86	3.79	3.87	0.11	*0.838*
C17:0	0.18	0.19	0.16	0.01	*0.076*
C17:1 *cis-9*	0.50	0.44	0.43	0.02	*0.094*
C18:0	11.40	11.37	11.77	0.22	*0.548*
C18:1 *trans-9*	0.230 ^a^	0.26 ^a,b^	0.22 ^b^	0.02	*0.002*
C18:1 *n-9 cis*	45.47	45.66	45.91	0.47	*0.806*
C18:2 n-6 trans	0.015	0.006	0.005	0.007	*0.106*
C18:2 n-6 cis	7.36	7.32	7.31	0.33	*0.994*
C18:3 n-6	0.10	0.10	0.08	0.01	*0.140*
C18:3 n-3 ALA	0.76 ^a^	0.76 ^a^	0.65 ^b^	0.03	*0.015*
C20:0	0.22	0.21	0.21	0.01	*0.686*
C20:1 *cis-9*	0.38 ^a,b^	0.42 ^a^	0.33 ^b^	0.03	*0.042*
C20:2 n-6	0.27	0.25	0.23	0.02	*0.206*
C20:3 n-6	0.27	0.26	0.24	0.02	*0.473*
C20:3 n-3	0.06	0.04	0.04	0.004	*0.122*
C20:4 n-6	2.04	1.90	1.98	0.14	*0.764*
C20:5 n-3 EPA	0.06	0.06	0.05	0.004	*0.347*
C22:0	0.12 ^a^	0.12 ^a^	0.09 ^b^	0.01	*0.005*
C22:1 *cis-9*	0.02 ^a,b^	0.04 ^a^	0.01 ^b^	0.006	*0.003*
C22:4 n-6	0.49	0.46	0.46	0.032	*0.802*
C22:5 n-3	0.27	0.34	0.32	0.023	*0.091*
C22:6 n-3 DHA	0.12 ^a,b^	0.13 ^a^	0.09 ^b^	0.01	*0.005*
C24:0	0.09	0.11	0.1	0.007	*0.506*
C24:1 *cis-9*	0.07	0.06	0.06	0.005	*0.829*
Σ SFAs	37.40	37.47	37.63	0.36	*0.894*
Σ MUFAs	50.76	50.90	50.92	0.48	*0.965*
Σ PUFAs	11.84	11.60	11.45	0.56	*0.880*
Σ n-6	10.54	10.30	10.30	0.52	*0.935*
Σ n-3	1.26 ^a,b^	1.30 ^a^	1.15 ^b^	0.04	*0.015*
n-6/n-3	8.35 ^a,b^	7.67 ^b^	8.98 ^a^	0.28	*0.006*

**Table 8:** EMM and SEM of fatty acids (FAs) as % of total FAs assayed in *Longissimus dorsi* of reared indoors + outdoor under different conditions of SA (Experiment 2). Within a row, different letters denote significant differences among sired lines.

	Space Allowance in Experiment 2—Indoor + Outdoor				
	1.4 + 1 m^2^/pig	2.6 + 2 m^2^/pig	3.9 + 3 m^2^/pig		
Samples (*n*.)	34	22	33		
	EMM	EMM	EMM	SEM	*p*
FAs (% total FAs)					
C10:0	0.14	0.14	0.13	0.007	*0.543*
C12:0	0.10	0.09	0.09	0.007	*0.601*
C13:0	0.11	0.12	0.09	0.02	*0.416*
C14:0	1.35 ^b^	1.51 ^a^	1.39 ^a,b^	0.04	*0.023*
C14:1 *cis-9*	0.10	0.10	0.07	0.02	*0.321*
C15:0	0.07	0.07	0.13	0.04	*0.269*
C15:1 *cis-10*	0.09	0.08	0.07	0.01	*0.375*
C16:0	22.47	22.72	23.42	0.31	*0.105*
C16:1 *cis-9*	3.59	3.43	3.59	0.09	*0.353*
C17:0	0.22 ^a,b^	0.23 ^a^	0.19 ^b^	0.01	*0.010*
C17:1 *cis-9*	0.52 ^a^	0.52 ^a^	0.45 ^b^	0.03	*0.020*
C18:0	10.93	11.16	10.91	0.20	*0.461*
C18:1 *trans-9*	0.27	0.27	0.26	0.02	*0.968*
C18:1 *n-9 cis*	45.39 ^a,b^	44.98 ^b^	46.11 ^a^	0.45	*0.051*
C18:2 n-6 trans	0.002	0.003	0.007	0.004	*0.651*
C18:2 n-6 cis	8.14 ^a^	8.27 ^a^	7.24 ^b^	0.32	*0.008*
C18:3 n-6	0.09	0.10	0.08	0.007	*0.076*
C18:3 n-3 ALA	0.82	0.81	0.81	0.03	*0.855*
C20:0	0.25	0.25	0.24	0.01	*0.866*
C20:1 *cis-9*	0.58	0.61	0.55	0.03	*0.294*
C20:2 n-6	0.29 ^a,b^	0.32 ^a^	0.27 ^b^	0.01	*0.006*
C20:3 n-6	0.29	0.30	0.26	0.02	*0.078*
C20:3 n-3	0.075 ^a,b^	0.083 ^a^	0.06 ^b^	0.005	*0.042*
C20:4 n-6	2.13 ^a,b^	2.20 ^a^	1.82 ^b^	0.11	*0.022*
C20:5 n-3 EPA	0.11 ^a^	0.12 ^a^	0.09 ^b^	0.006	*0.008*
C22:0	0.13	0.14	0.13	0.008	*0.436*
C22:1 *cis-9*	0.02	0.02	0.02	0.006	*0.591*
C22:4 n-6	0.49	0.49	0.44	0.031	*0.292*
C22:5 n-3	0.47 ^a^	0.49 ^a^	0.39 ^b^	0.027	*0.022*
C22:6 n-3 DHA	0.15 ^a^	0.12 ^b^	0.12 ^b^	0.01	*0.013*
C24:0	0.11 ^a^	0.11 ^a,b^	0.09 ^b^	0.007	*0.017*
C24:1 *cis-9*	0.08	0.09	0.07	0.005	*0.036*
Σ SFAs	35.98	36.71	37.16	0.35	*0.064*
Σ MUFAs	50.95 ^a,b^	49.94 ^b^	51.23 ^a^	0.41	*0.042*
Σ PUFAs	13.05 ^a^	13.31 ^a^	11.61 ^b^	0.45	*0.012*
Σ n-6	11.43 ^a^	11.68 ^a^	10.14 ^b^	0.41	*0.013*
Σ n-3	1.62 ^a^	1.63 ^a^	1.47 ^b^	0.05	*0.004*
n-6/n-3	7.05	7.17	6.89	0.18	*0.217*

## Data Availability

The data presented in this study are openly available in FigShare at https://doi.org/10.6084/m9.figshare.29437430 (accessed on 30 June 2025).

## Abbreviations

The following abbreviations are used in this manuscript:

AW	Animal Welfare
SA	Space Allowance
FA	Fatty Acid
SFA	Saturated Fatty Acid
MUFA	Monounsaturated Fatty Acid
PUFA	Polyunsaturated Fatty Acid
ALA	Alpha-Linolenic Acid
EPA	Eicosapentaenoic Acid
DHA	Docosahexaenoic Acid
PDO	Protected Designation of Origin
BW	Body Weight
ADG	Average Daily Gain
FCR	Feed Conversion Ratio
SSF	Slice Shear Force
GLM	General Linear Model
EMM	Estimated Marginal Mean
SEM	Standard Error of Mean
